# Some recommendations for developing multidimensional computerized adaptive tests for patient-reported outcomes

**DOI:** 10.1007/s11136-018-1821-8

**Published:** 2018-02-23

**Authors:** Niels Smits, Muirne C. S. Paap, Jan R. Böhnke

**Affiliations:** 1 Research Institute of Child Development and Education, University of Amsterdam, Nieuwe Achtergracht 127, 1018 WS Amsterdam, The Netherlands; 2 Department of Special Needs, Education, and Youth Care, Faculty of Behavioural and Social Sciences, University of Groningen, Groningen, The Netherlands; 3 Dundee Centre for Health and Related Research, School of Nursing and Health Sciences, University of Dundee, Dundee, UK

**Keywords:** Multidimensional item response theory, Computerized adaptive testing, Item bank, Patient-reported outcomes, Quality of life

## Abstract

**Purpose:**

Multidimensional item response theory and computerized adaptive testing (CAT) are increasingly used in mental health, quality of life (QoL), and patient-reported outcome measurement. Although multidimensional assessment techniques hold promises, they are more challenging in their application than unidimensional ones. The authors comment on minimal standards when developing multidimensional CATs.

**Methods:**

Prompted by pioneering papers published in QLR, the authors reflect on existing guidance and discussions from different psychometric communities, including guidelines developed for unidimensional CATs in the PROMIS project.

**Results:**

The commentary focuses on two key topics: (1) the design, evaluation, and calibration of multidimensional item banks and (2) how to study the efficiency and precision of a multidimensional item bank. The authors suggest that the development of a carefully designed and calibrated item bank encompasses a construction phase and a psychometric phase. With respect to efficiency and precision, item banks should be large enough to provide adequate precision over the full range of the latent constructs. Therefore CAT performance should be studied as a function of the latent constructs and with reference to relevant benchmarks. Solutions are also suggested for simulation studies using real data, which often result in too optimistic evaluations of an item bank’s efficiency and precision.

**Discussion:**

Multidimensional CAT applications are promising but complex statistical assessment tools which necessitate detailed theoretical frameworks and methodological scrutiny when testing their appropriateness for practical applications. The authors advise researchers to evaluate item banks with a broad set of methods, describe their choices in detail, and substantiate their approach for validation.

## Introduction

Multidimensional applications of item response theory (IRT) are seen as a somewhat theoretical—albeit desirable—addition to the toolkit of QoL researchers [1–3]. However, the number of studies published in Quality of Life Research taking advantage of the unique properties of multidimensional IRT has been increasing [4–6], and so has the interest in developing multidimensional computerized adaptive testing (CAT) applications [7, 8]. These developments are very welcome. First, although there is little agreement as to which domains form the construct of “(health-related) QoL,” it is likely to be multidimensional or multifaceted [9]. Psychometric methods incorporating this potential multidimensionality are therefore an obvious choice to connect theoretical concepts and empirical research. If one decides to take multidimensionality into account, several options can be considered. For example, Adams and colleagues [10] divide multidimensional IRT models into two subclasses: within-item and between-item models, which correspond to the ‘complex’ and ‘simple’ structures in factor analysis [11, 12]. Within-item multidimensional models allow for more than one discrimination parameter per item. When between-item multidimensional models are used, the items are “assigned” to one dimension only and multidimensionality is modeled through the correlations among the latent dimensions. Second, two conceptually related methods have been key staples of QoL research so far: (1) unidimensional CAT applications to balance assessment burden and precision, as well as (2) factor analytic approaches to explore the dimensionality of measurement instruments. Multidimensional CAT could be seen as an approach that unifies key elements of both CAT and factor analytic approaches—which typically account for multidimensionality—and can supplement these widely used approaches [13]. And third, convincing evidence from statistical research in clinical trials both within classical as well as modern test theory suggests that increasing reliability of assessment instruments increases the statistical power of studies [14–18]. As CATs take the reliability of the individual’s test score continuously into account until a minimally required level of measurement precision has been attained, compared to fixed-form measurement instruments, they provide an opportunity to increase the quality of outcome assessment in clinical studies.

Given these arguments, a multidimensional CAT perspective has a lot to offer for the assessment of reflective constructs [19] and a discussion of methodological approaches may be timely [13, 19]. After all, just fitting an IRT model to a set of item responses does not mean that one has established an item bank, let alone measurement of a latent variable. In this commentary, we therefore reflect on current practices in the field of QoL research, revisit existing recommendations regarding test and CAT development, and review whether these recommendations are generalizable to multidimensional applications and to QoL outcomes. Our commentary presents suggestions for minimal standards in the development in multidimensional CAT, and is organized in two sections: (1) the design, evaluation, and calibration of multidimensional item banks; and (2) how to study the efficiency and precision of a validated item bank.

## Item bank design

Multidimensional CATs should draw from a carefully designed and calibrated multidimensional item bank. A frequently employed strategy to develop CAT is to use a single or limited number of pre-existing tests or questionnaires [7, 8], while others extended work on existing item banks [6]. A disadvantage of using such pre-existing item banks, and especially fixed-form questionnaires, is that the legacy measures in question were not designed to be used as multidimensional item banks. *We therefore advocate the use of a thorough item bank development process, consisting of both a test construction phase and a measurement property/psychometric phase*. In the following, we highlight important decisions in both phases.

### Construction phase

Test development usually starts with a definition or description of the construct(s) for which items will be created and several conceptual frameworks have been proposed to provide item writers with guidelines for that step [20]. For example, one can use a *deductive* strategy in which items are derived from an explicit theory about the construct, its constituting aspects, and its relation with other constructs [21]. Alternatively, one can take an *intuitive* approach in which a description of the construct is based on the intuitive knowledge of experts and patients [22]. Each strategy has its merits. The deductive strategy allows for a balanced representation of the content domain; intuitive methods usually lead to items that are aligned with the experience of both experts and patients. In practice, item writing will take ingredients from both strategies, and which strategy prevails depends on the availability of theories about the content domain and test goal of the anticipated item bank.

After specifying the content domains, the next step consists of writing and selecting individual items for each domain. If relevant items are available from existing instruments, permission should be obtained to use these items. The final set of items should allow for targeting a broad range of trait levels, and therefore existing sets of items may need to be extended with new items. When developing a multidimensional item bank, the items should be constructed and/or selected in such a way that they follow the assumed multidimensional structure (see below).

The next step is to perform a pilot study to assess the quality of the provisional item pool. In this pilot study, the items are presented to and reviewed by two target groups [20]: (a) experts (for example [23]) and (b) patients (for example [22]). Based on these reviews, items may be revised or removed from the pool resulting in a first draft of the item bank, which is then administered in the calibration sample to evaluate its psychometric properties.

### Psychometric evaluation and calibration

Reeve and colleagues [24] suggested a detailed plan for psychometric evaluation and calibration for unidimensional CATs. They distinguish five steps: evaluating traditional descriptive statistics, evaluating assumptions of the IRT model, fitting the IRT model, evaluating differential item functioning for key demographic and clinical groups, and finally item calibration for item banking. Although the five steps by Reeve and colleagues are specific for the PROMIS framework [24], they form a suitable starting point for the development of other health-related CATs. In the following, we highlight where the five steps may need to be extended for the multidimensional case, or prove more difficult to follow than in a unidimensional context.

A key choice in the “psychometric phase” of item bank development is which IRT model to use. As in the unidimensional case, it is important to consider how many item parameters need to be included in the model, whether the response process follows a dominance or an ideal point process [25], which link function to use (logistic/normal ogive), how to treat item category responses (nominal/dichotomous/polytomous), and in case of polytomous items how to model category responses (e.g., using a generalized partial credit model, sequential model, or graded response model). And since each of these choices impacts the plausible link between the latent trait and question responses, some of these choices should already inform question development in the construction phase.

If there is multidimensionality present in the data, additional choices need to be made. First, one needs to decide whether or not to ignore the multidimensionality. Some prefer to apply unidimensional models when a set of items is deemed “unidimensional enough,” while others would reason that constructs such as health-related QoL are multidimensional by definition and ignoring this multidimensionality leads to measurements of lower quality. To a degree then, this is a matter of preference. A decision may be based on interpreting psychometric results (such as the proportion of explained variance attributable to the main factor [2, 26]), on substantive or theoretical grounds, on practical considerations (interpretability, acceptability to end-users), or a combination of two or more of the aforementioned. The effect of ignoring multidimensionality on parameter estimates is different for between-item and within-item multidimensionality [27]. Ignoring between-item multidimensionality is likely to have a negative impact on measurement precision, but the impact on parameter point estimates is typically small. Ignoring within-item multidimensionality, on the other hand, may lead to bias in the parameter estimates. For a discussion of psychometric methods of determining the multidimensional structure of test data, the reader is referred to Reckase (Chap. 7 in [28]).

As mentioned earlier, multidimensionality can be modeled in different ways. A key difference is whether the model allows for between- or within-item multidimensionality. Another relevant distinction is whether or not a hierarchy at the latent trait level is introduced into the model. In second-order factor models, first-order factors are distinguished from a general factor; the general factor is used to explain the association between the first-order dimensions. In contrast, bifactor models explain all observed item scores with a general factor, but in addition group factors are specified which capture the covariance among groups of items independent of the general factor (e.g., content domains; [2]). Although bifactor models have become very popular recently, they are not always appropriate and need to be carefully evaluated in every application [26].

Which type of model to favor may depend on a number of different considerations. Some may simply use the best-fitting model, whereas other may prefer to choose a model which is closely aligned with their theoretical model. The former approach may lead to capitalization on chance [29] and therefore it may be wise to perform some sort of cross-validation [30]. *In our opinion, there is no definitive answer to the question how one can determine what the best multidimensional IRT model is in a given situation; what is important, is that the authors clearly describe what model they used (preferably by including a mathematical statement) and explain why they favored this particular model, based on theoretical as well as empirical grounds*.

Although multidimensional IRT and CAT may offer substantial benefits for assessing QoL, methodological knowledge and software development lag behind, especially when compared to what is available for the unidimensional case. For example, although Reeve et al. [24] provide several heuristics and validation steps for developing unidimensional CATs, it is yet unclear if they are also appropriate in the multidimensional case. Currently available software packages for estimating multidimensional IRT models [31–34] have some limitations; as a result, researchers may be required to use multiple packages when evaluating the psychometric properties of the item bank. For example, the type of models supported differs per package; furthermore, in case of large numbers of missings, item fit statistics are unavailable or do not work properly in some packages; and when the number of dimensions gets large, advanced numeric estimation techniques fail. *It is therefore advised that authors provide an overview of the limitations of their study due to unsettled issues in both methodology and software*.

And finally, when estimating the multidimensional IRT model underlying the item bank, an appropriate validation sample should be used [35]. First, the sample should be taken from the population for which the multidimensional CAT will be implemented so that the latent trait is scaled with reference to this population. Second, to obtain precise estimates of the item parameters, the sample should contain individuals of varying latent trait values so that all item options are selected a substantial number of times. It may therefore be necessary to include additional respondents who are known to have extreme scores [35]. *In such cases it is very important that authors appropriately incorporate extra observations into the model (either by linking or weighting) because it has been shown that failing to do so may lead to bias in item and person parameters, impacting population norms and CAT score reliability* [36].

In addition, the calibration sample should be large enough to provide precise estimates of the item parameters. For unidimensional IRT models, simulation studies have been performed examining the minimally required sample size, and these have suggested sample sizes of the order of 500–1000 appropriately chosen respondents [37, 38]. Similarly, simulation studies suggest that for multidimensional IRT models sample sizes should be at least 500 in most cases, and 1000 in case of large item banks [39, 40]. Although the outcomes of these studies are very valuable, their conclusions are only valid in a limited set of conditions, such as a three-dimensional model, multivariate normally distributed latent traits, a between-item multidimensional structure, an equal number of items for each dimension, and item banks with a uniform measurement precision in the latent trait space. Simulation studies in unidimensional CATs have shown that deviations from standard conditions generally lead to higher requirements with reference to sample size for stable calibration (i.e., parameter estimates coming close to true parameter values), and, in all likelihood, this is true for the multidimensional case as well. *In many empirical applications, less favorable conditions may be encountered, and for such situations authors are advised to be conservative, aiming for at least 1000 rather than 500 observations for sound item calibration*.

## Efficiency and precision of an item bank

CAT is often claimed to solve issues in the clinical field such as scoring questionnaires by hand and responder burden. However, constructing, implementing, and maintaining a CAT in the real world is an enormous investment, and the reports on the construction of new item banks should therefore provide evidence of a large improvement in efficiency. Early studies comparing multidimensional to unidimensional CAT efficiency focused on educational achievement. These studies showed that multidimensional CATs were 25–33% shorter [41–43]. Two recent studies [44, 45] in the context of health measurement showed that the efficiency gains reported for achievement measurement seem to generalize to health measurement: between-item multidimensional CATs were on average 20–38% shorter compared to using separate unidimensional CATs when between-dimension correlations were high (*r* > .76). For weaker correlations (*r* = .56), multidimensional CATs were on average 17% shorter than unidimensional CATs [45].

Item banks should contain items with high-quality measurement properties, and there should be enough of such items in the bank. A popular rule for stopping a CAT-assessment in both the unidimensional [46] and multidimensional [47] case is when measurement precision is high enough. In IRT, measurement precision is a function of the latent trait. As a consequence, specific items may lead to a larger increase in measurement precision for some patients compared to others; for example, for a patient with a high score on the construct, an item may measure the construct with little precision, whereas the same item may measure the construct with a high degree of precision for a patient with a low score. By definition, the measurement precision an item bank provides is the sum of the measurement precision of its constituting items. *Therefore the presented item bank should be large enough to support adequate measurement precision for all relevant levels of the latent construct one wishes to assess in the given application* [48, 49]. Past studies suggest that item banks as small as 20–30 polytomous items per dimension may be adequate when exposure control is not an issue [50, 51]. A recent study [45] found that the required item bank size depended on adequate targeting (the match between the item bank and the latent trait distribution). When targeting was spot-on, five items per dimension would suffice to support CAT. In contrast, when targeting was problematic, as many as 120 items per dimension were needed. Multidimensional CAT generally resulted in a higher proportion of individual CAT assessments reaching the required measurement precision threshold. These findings indicate that the required bank size depends on the CAT algorithm used (uni- or multidimensional) and on bank information. The required bank size is smaller for multidimensional CATs, and for banks that contain more information for the latent trait-range of interest. *We therefore recommend that these factors be taken into consideration when making a final decision regarding bank size*.

To quantify measurement precision of latent trait scores, IRT often uses the standard error (*SE*) of the latent trait estimate. Often, to improve interpretability, *SE* is translated into the scale of traditional reliability which runs from 0 to 1; for example, an *SE* of 0.22 (when the latent trait is assumed to follow a *N* [0, 1] distribution) corresponds to a reliability index of 0.95 [49]. For traditional reliability, heuristics have been suggested for specific testing situations. For example, if important decisions are made with respect to an individual’s test score, a reliability of 0.90 has been claimed to be the bare minimum [52], which would come down to a minimal SE of 0.31. If making individual decisions is the assessment goal, the item bank developer should make sure that the item bank allows for *SE*s of at least 0.31 along the full latent trait continuum for broad use, or at least in those trait ranges that are relevant in the specific application. In the multivariate case, it is harder to evaluate the measurement precision of an item bank, because (i) multidimensional measurement precision is conceptually harder to understand, and (ii) graphical methods for displaying measurement precision have not yet been developed for item banks with more than two dimensions [28]. *Therefore, in the multidimensional case it is advised to evaluate measurement precision for each dimension separately* [35], *or to perform simulations to map the measurement precision of an item bank* [46].

In multidimensional CAT simulation studies, two outcome variables are of importance: efficiency and precision. Like for regular CAT [53], the *efficiency* of multidimensional CAT may be quantified using descriptive statistics of the number of items used; for overall efficiency, the average or median may be used; to investigate the variability over administrations, the standard deviation or the range may be used. Compared to regular CATs, multidimensional CATs use item banks that are arranged into domains, dimensions, or facets, and often it is important that each part is equally covered in the adaptive test [8]. *In such cases, authors should also provide efficiency results for each level of these arrangements separately*.

To express the precision of a unidimensional CAT administration, several *outcome variables* have been suggested, for example, the correlation between the true and estimated latent trait [54] and the root mean squared difference between these values [55]. The latter statistic is expressed on the same scale as *SE*, and may therefore be used to compare nominal and real precision; for audiences with less technical knowledge these outcomes may be translated in terms of traditional reliability. In addition, to examine if the CAT algorithm generates structural deviations between true and estimated latent trait values (i.e., bias), the average difference may be used. The latter outcome is often used to study the impact of a chosen method for estimating the latent trait (such as maximum a posteriori). As noted above, for multidimensional CAT, a choice must be made how to deal with multidimensional nature: outcomes may be studied either for each dimension separately, and/or appropriately aggregated over dimensions [47, 56].

*Because the requirement for CATs is often that measurement precision and efficiency are high enough over all relevant levels of the latent trait, simulation outcomes should be presented as a function of the latent trait values*. For multidimensional CAT, this may be a bit of a challenge because with increasing number of dimensions the number of combined levels of the latent traits increases exponentially; a solution would be to use fewer values per dimension, or group combinations of latent trait values into categories.

For multidimensional CAT simulation two types of data can be used as input: real and simulated data. Both types have advantages and disadvantages, and the advantage of the one type is a disadvantage of the other. An advantage of real data is that they are usually readily available, such as the item responses of the calibration sample; although software is available to do this automatically for calibrated models [32] simulated data take time and effort to produce. In addition, whereas results from real data are convincing to the audience because they show what may be experienced in practice, results from synthetic data may seem less relevant because they are obtained in the somewhat improbable situation of the data perfectly fitting the calibrated model. A disadvantage of using real data is that true latent trait levels are not available. Often, the latent trait estimate based on the full item bank is treated as a proxy for the true latent trait value, and the final score from the adaptive test is used as the estimated value [57]. *We recommend that scholars using real data simulations acknowledge this limitation, and stress that the statistics they calculate using full bank estimates rather than true latent trait values are proxies*.

Moreover, using real data can easily lead to results that are too optimistic, especially when the number of observations is small. Using a sample for both item calibration and studying CAT performance may lead to overfitting ([58], i.e., capitalizing on chance): due to sampling fluctuations, parameter estimates generally vary over samples, and the estimated item parameters of a given sample are the best fitting ones for that sample, but not for other samples. As a consequence, the similarity between true and estimated latent traits for respondents from that sample will be higher than for respondents not in that sample (such as future users), and reporting this similarity will therefore overestimate the actual performance of a CAT. A possible solution for overfitting would be to perform cross-validation in which the original sample is split into two sub-samples; the first sample is used to estimate the structural parameters, which are then put to use in the second to evaluate efficiency and precision in a CAT simulation [59]. *It is therefore advised that authors acknowledge the possibility of overfitting and provide an appropriate solution for it*.

Another reason for real data possibly giving optimistic outcomes is that estimates of the latent traits based on the full item bank and those based on a CAT use overlapping item responses, and therefore the two sets of estimates would be expected to correlate even if the item scores would have been produced randomly [60]. *It is therefore advised to also report the correlation between the CAT-based estimates and estimates based on items that were not administered in the CAT*.

*Finally, in order for the research community to be able to properly evaluate the gain in efficiency of a new CAT, relevant benchmarks should be provided*. For example, when evaluating a unidimensional CAT, one may also provide the precision of a static short form. When evaluating a multidimensional CAT, one may also provide the outcomes of combined CATs for each separate dimension.

## Conclusion

This commentary aimed at highlighting challenges when developing multidimensional item banks for patient-reported outcomes. Based on previously published frameworks and recent research, we provided suggestions for minimal standards for future ventures into this area, addressing both (1) the design, evaluation, and calibration of multidimensional item banks and (2) how to study the efficiency and precision of a validated item bank. It may be worthwhile noting that (2) is only useful if (1) has been completed successfully, and the psychometric properties of the item bank have been found acceptable for the target population.

We did not provide further detail on several related topics beyond the focus of our commentary. First, we assumed some familiarity with IRT and CAT, and refer to a number of introductory texts available for the interested reader [35, 61, 62]. Second, we focused on how to build an empirical argument that an item bank has a presumed structure and measures one or more constructs. We did not discuss how to establish that it measures what it is supposed to measure (i.e., has adequate validity). This key quality criterion for any assessment measure needs to be demonstrated separately, for example, covering all relevant content [63], predicting external criteria [64], and fitting into the relations posed by the bank’s nomological net [65]. Third, item banks and CAT rest on the assumption that one or more latent variables determine the responses to the items (i.e., reflective measurement; [19]). This assumption may not be tenable, for example, for items that constitute a composite index (i.e., formative measurement; [66–68]), or items that were calibrated according to population preferences (preference-based measurement; [69]). At the moment no framework unites these different approaches. Fourth, since the Food & Drug Administration’s publication of guidance regarding the use of patient-reported outcomes (PROs [70, 71]) they are discussed more widely [72–75]. The reliability of such measures, their content coverage, their appropriateness for the target population, and burden associated with PRO assessments are recurring topics and multidimensional IRT and CAT can enrich these debates. Finally, we built on previously published checklists for developing item response models for PROs [76] and unidimensional item banks [24], and our comment can be seen as an addition to these. An overview of our suggestions is presented in Table 1, summarizing our line of argument.

**Table 1:** An overview of recommendations for the development of CATs

1	The item bank development process should consist of *both* a test construction phase and a psychometric evaluation and calibration phase.
2	Providing an extensive account of both theoretical and empirical grounds for choosing a specific uni- or multidimensional IRT model is essential
3	An overview of the limitations due to unsettled issues in both methodology and software should be provided
4	Observations with extreme scores, added to the calibration sample to increase the precision of item parameter estimates, must be properly incorporated into the IRT model
5	In the calibration phase of multidimensional IRT models, it is advised to be conservative, aiming for at least 1000 observations
6	The presented item bank should be large enough to support adequate measurement precision for all relevant levels of the latent construct(s)
7	If some knowledge of both item information and dimensionality is available in advance, it is advised to also take it into account when deciding on how many items should be included in item bank (more information and more dimensions allow for fewer items)
8	To map measurement precision in the multidimensional case it is advised to either evaluate it for each dimension separately or to perform simulations in the multidimensional space
9	When studying efficiency of an item bank in case of separate dimensions, domains, or facets, results should be presented for each level of these arrangements separately
10	When studying efficiency and accuracy of an item bank, outcomes should be presented as a function of the latent trait
11	When the validity of an item bank is studied using real data simulations, it should be acknowledged that full bank estimates are not true values of latent traits, but proxies
12	When the efficiency of an item bank is studied using real data, the possibility of overfitting exists and an appropriate solution should be chosen to prevent it
13	When comparing full item bank estimates and CAT estimates, high congruence is expected because both are partly based on the same data. It is therefore advised to also report the association between the CAT-based estimates and estimates based on unadministered items
14	When evaluating the efficiency of a new CAT, relevant benchmarks, such as results using short forms or results per dimension should be provided

As statistical methods get more complex, we acknowledge that not all suggestions may be relevant in all applications. Furthermore, in some cases, it may not be feasible to satisfy some of the criteria; here, we encourage authors to explain why certain steps were omitted. As recently suggested by Sprangers and Schwartz [77], QoL research has developed a broad, rigorous, and diverse set of methods which allow for a comprehensive investigation of relevant phenomena. Nevertheless, they also note “[w]hile this maturation is laudable and needed, it can result in a limiting rigidity.” [77, p. 1387] In the spirit of their article, we do not wish this commentary to be seen as yet another addition to an ever expanding collection of rules that potentially hinder the discovery process. On the contrary, we hope that our discussion adds to the pioneering papers in an area that is in our opinion likely to become a key methodological building block of quality of life research. We hope that this commentary helps researchers and practitioners to evaluate the potential and relevance of multidimensional CAT for their applications.
